# The American and Colonial Journals: Anatomy and Physiology

**Published:** 1842-10

**Authors:** 


### II. THE AMERICAN AND COLONIAL JOURNALS.

### ANATOMY AND PHYSIOLOGY.

*Evolution of Electricity.* By Dr. William Muller.

The author details some very curious results of experiments made by him, of which the following particulars are the principal:

Place the electrometer on the mantel-piece over a good fire. Take a common sized chair, with a back to it, of such a height, that the feet resting on the floor, the thighs shall be horizontal. Sit towards the front edge of the chair, and lean back, so as to have the trunk of the body quite relaxed; then rise quickly and touch the cover of the electrometer. The leaf, or leaves, will scarcely fail to indicate the presence of eletricity. If the first trial should fail, it will be owing to the non-observance of some of the above mentioned conditions; a second or third *must* succeed. The electrometer may also be placed on a table before the fire; the experimenter seated, as described, on a chair near it, may place his hand on the cover, and then, after leaning back, he should lean a little forwards and rise quickly, or but partially assume the erect position. At the instant of rising, and very often at that of sitting down after having risen thus, the electrometer will indicate a large amount of electricity. I have charged a jar with as much as could be detected by the instrument, by thus alternately rising and sitting. I have not, however, been able to cause the leaf to move more than a quarter of an inch by applying a jar thus charged by half a dozen risings and sittings; though, by keeping the finger on the electrometer while I thus rose and sat, I could, as I said before, cause a continual flight of the leaf to and fro through an inch and more. I have hitherto found my own electricity positive, and I have a suspicion that the electricity is different according as I rise up or sit down. This shall be decided by future experiments.

Prevost and Dumas pretend to have shown that electricity is produced during muscular contraction, and Edwards has shown that the same bodies which do or do not conduct electricity, do or do not conduct the nervous power, but no one till now, has observed the relation which exists between bodily motion in a particular direction, and a copious evolution of electricity.

*Medical Examiner.* No. viii. *Feb.* 19, 1842.

